# Multiorgan failure with fatal outcome after stem cell tourism

**DOI:** 10.1186/s40001-020-00477-4

**Published:** 2021-01-09

**Authors:** Željka Večerić-Haler, Špela Borštnar, Boštjan Luzar, Maja Jerše, Nika Kojc

**Affiliations:** 1 Department of Nephrology, University Medical Centre Ljubljana, Ljubljana, Slovenia; 2 Institute of Pathology, Faculty of Medicine, University of Ljubljana, Ljubljana, Slovenia; 3 Faculty of Medicine, University of Ljubljana, Ljubljana, Slovenia

**Keywords:** Stem cell, Stem cell tourism, Unproven stem cell treatment, Hepatitis, Skin ulcer, Calciphylaxis, Cardiomyopathy, Multiorgan failure

## Abstract

**Background:**

Unproven stem cell treatments may involve serious health, personal, and financial considerations. Due to worldwide spread, illegal stem cell therapies have become a major public health problem. We have already witnessed numerous reports in the mass media of severe and occasionally even fatal outcomes after such therapies. However, there are only few scientifically documented cases in which the causality between stem cell therapy and side effects cannot be refuted.

**Case presentation:**

Here we present a case report of a 48-year-old patient with serious side effects, including disseminated skin ulcers, hepatitis, and cardiomyopathy, with eventual fatal outcome following unproven stem cell treatment.

**Conclusions:**

The case of the patient presented here draws attention to the worst possible outcome of stem cell tourism. To effectively combat this issue, professionals and patients should be empowered with the right knowledge on possible side effects.

## Introduction

Patients suffering from various serious conditions can become desperate when conventional medicine fails to provide a solution for their disease. In recent years, there have been a growing number of predatory clinics that have begun advertising unproven therapies at great cost to the patient—this is now referred to as “stem cell tourism” [1]. Many advertised treatments are clinically unproven, frequently presented as being helpful in most debilitating conditions, with little or no evidence of their actual safety and efficacy. Most of such treatments are autologous cellular therapies (patients' own cells are used), although some clinics also claim to use allogenic cord blood, or embryonic and fetal cells. However, it is hard to know what is actually being administered to recipients. There are reports of severe side effects and even fatal outcomes after such unregulated interventions [2]. Herein, we present a case report of a patient with serious side effects and eventual fatal outcome following unproven stem cell treatment.

## Case report

A 48-year-old man with end-stage kidney failure, on maintenance dialysis since 2011, was referred in February 2020 with a 6-month history of general malaise and progressive evolvement of painless icterus and necrotizing skin ulcers.

The patient’s medical history included diabetes mellitus (DM) type I with end-stage kidney failure, ischemic cardiomyopathy (obstructive lesions of left anterior descending coronary artery resolved by stent placement in 2018), and signs of predominantly diastolic heart failure (ultrasound of the heart prior to the occurrence of the symptoms referred to herein showed a 49% ejection fraction, mild mitral and aortic regurgitation, and moderate pulmonary hypertension with 47 mmHg). His official therapy consisted of lacidipine, furosemide, acetylsalicylic acid, calcium carbonate, bisoprolol, sevelamer, and pantoprazole, and was substantially unchanged in the last year.

In December 2018, the patient underwent stem cell transplantation in Ukraine. The stem cell clinic NBS/ICH/Kiev/London promised––as cited from the brochure, with many grammatical errors»…it is expected strengthening of the regenerative abilities of the body, improving of all functions of organs and tissues and repairing of pathologically damaged tissues, rejuvenate body…« The patient explained he mostly hoped for restitution of his kidney function. Although the patient could not provide an exact operative report, he explained that the product was supposed to be stem cells of embryonal origin and was injected in a single infusion in his peripheral vein. The stem cell clinic claimed in their informative material that examination (including determination of normal heart, lungs, kidney and liver function, as well as ruling out infection) would be performed. However, the patient could not recall any preadmission testing.

**Fig. 1:**
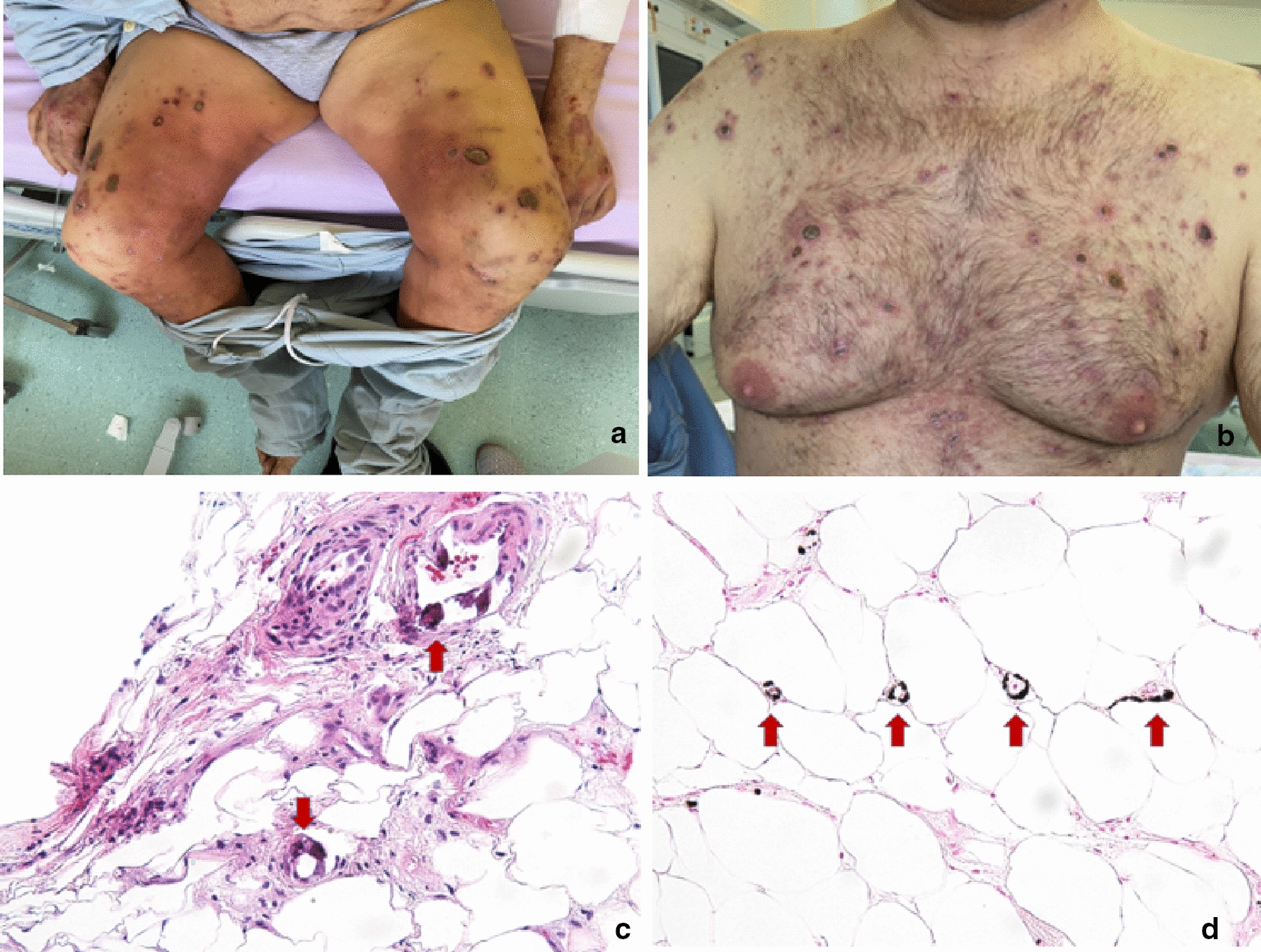
**a**, **b** Necrotizing skin ulcers on the patient’s legs and trunk; **c** Skin biopsy. Basophilic calcification in smooth muscle cells of subcutaneous artery and arteriole (HE, original magnification, 200 ×); **d** von Kossa stain highlights subtle calcium deposits in arterioles and also in the interstitium of the subcutis (original magnification, 200 ×). Calcifications around vessels and in the interstitium are marked with arrows

At the current referral, jaundice (bilirubin 142/116 μmol/l, increased alkaline phosphatase, slightly elevated alanine aminotransferase) and necrotizing skin changes predominated in the clinical picture (Fig. 1a, b). He described his skin wounds as aching and itching. The first skin lesions appeared 6 months after stem cell treatment, starting as bullae, which then burst, and a scab formed underneath. The wounds spread all over the body, the most severely affected being the skin on the arms and legs. A deep-punch skin biopsy disclosed segmental medial basophilic calcification with focal atrophy of smooth muscle in media, intimal fibroplasia of small- and/or medium-sized arteries and arterioles in subcutaneous adipose tissue consistent with calciphylaxis. Additional staining with von Kossa highlighted subtle calcium deposits also in the interstitium of the dermis and elastic fibers. Inflammation was relatively scant as well as extravasation of erythrocytes. There were no changes suggestive for graft versus host disease (Fig. 1c, d).

However, the pathohistological diagnosis of calciphylaxis was not consistently supported by clinical findings, as there was no severe deterioration of phosphate/calcium metabolism on admission, parathyroid hormone was within limits, and, according to the angiologist's clinical assessment and ultrasound Doppler measurements of perfusion pressure in the lower extremity arteries, macrovascular peripheral arterial disease was not likely.

**Fig. 2:**
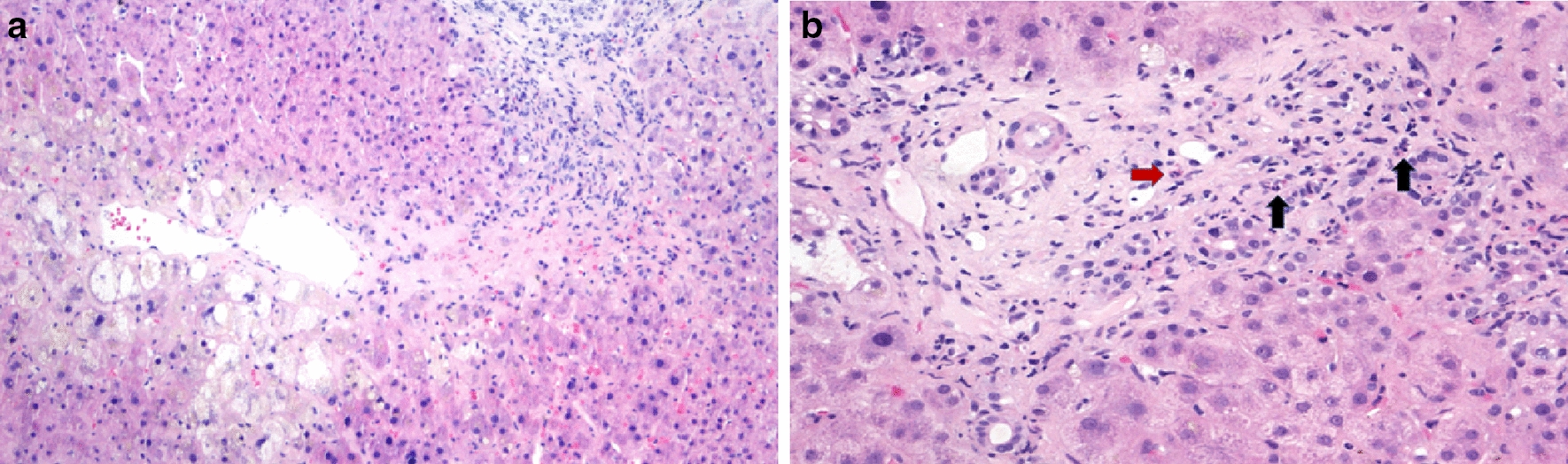
**a** Subacute hepatitis with the drop-out of hepatocytes, associated with collapse of hepatic parenchyma, ballooning degeneration of hepatocytes, moderate hepatocellular cholestasis, and mild mixed portal inflammation (HE, original magnification 100 ×). **b** Liver biopsy, higher magnification of a portal tract. A mixed inflammatory cell infiltrate composed of eosinophils (red arrow) and neutrophils (black arrows) is clearly apparent

The first laboratory signs of icterus were present in September 2019. Investigations of icterus included transabdominal and endoscopic ultrasound, which showed hepatosplenomegaly without signs of extrahepatic cholestasis. The most likely infectious, metabolic and autoimmune causes, as well as biliary obstruction (see Additional file 1: Table S1) were ruled out. Since the patient also experienced severe deterioration of heart function in the same time frame (heart ultrasound revealed a severe reduction of the left ventricular ejection fraction to 30%, segmental contraction disorders, right-sided heart failure, and moderate mitral regurgitation), the differential diagnosis included liver congestion due to heart failure of unknown etiology (acute coronary syndrome was ruled out). This was not, however, supported by the liver histology (Fig. 2a, b), which revealed subacute hepatitis with portal inflammation of mixed type (mainly neutrophilic and eosinophilic granulocytes) without convincing signs of chronic liver congestion. We concluded that the hepatic impairment was probably the result of combined liver defect: chronic congestive hepatopathy (dilated sinusoids and ultrasound dilated hepatic veins) and toxic injury of idiosyncratic type, which manifested as cholestatic hepatitis, revealed by liver biopsy. A reasonable trigger for this reaction was, however, unclear and difficult to identify.

In accordance with assumed calciphylaxis, treatment with sodium thiosulfate, intensified dialysis, and hyperbaric oxygen were initiated. The inflammatory parameters decreased, and the patient was cardiopulmonary compensated, with better but not normal laboratory results (bilirubin 45/45 μmol/l) and with slight improvement in skin condition. He insisted on dismission and continued with regular dialysis thrice weekly at our outpatient service. Four days after dismission he was found dead in his apartment. An autopsy was not performed.

## Discussion

The reported case describes a 48-year-old patient who developed necrotizing skin ulcers (pathohistologically confirmed as calciphylaxis), cholestatic hepatitis, and deteriorating heart failure in the period of 6 months post stem cell transplant at a commercial clinic in Ukraine. He was desperately seeking a cure for end-stage kidney failure and DM type I and the aforementioned clinic had offered an unrealistic assurance that stem cells could improve his health state. He underwent intravenous infusion of stem cells of unknown origin and, according to the patient’s report, was not given any immunosuppressive therapy prior to stem cell administration.

Although the patient had a number of pre-existing diseases, we were not able to explain and meaningfully connect his new-onset health problems and medical findings with other pre-existent conditions. After exclusion of infection and metabolic causes, toxic damage of idiosyncratic type, which could manifest as cholestatic hepatitis [3, 4] and various types of dermatitis [5], was discussed as a possible diagnosis.

In consideration of the fact that the patient did not have other newly introduced medications, in our opinion this complex multisystem disorder could be associated with the applied non-autologous stem cell product, stem cell medium, or both. Idiosyncratic reactions associated with various substances are characterized by a variable delay or latency period, ranging from a few days to several months from initial exposure to the agent. However, the majority of drug-induced liver injury cases show a steady decline in liver biochemistries after the presumed culprit agent is stopped [3, 4]. This observation, which is often referred to as “de-challenge” and is a major factor in diagnostic scoring algorithms of drug-induced liver injury, was not observed in our case. Another possibility that we additionally addressed was chronic graft versus host disease (GVHD), a common complication following allogeneic hematopoietic cell transplantation [6]. GVHD may involve a single organ or several organs, but typically manifests as injury to the skin, gastrointestinal mucosa, and liver. In some cases, hepatic GVHD may be histologically indistinguishable from other disorders, such as infection and drug-induced liver injury [7]. A skin biopsy, however, did not confirm histology lesions typical of cutaneous GVHD (interface dermatitis with vacuolar degeneration and lymphocyte satellitosis) [8], but revealed calciphylaxis.

There are several proposed risk factors and triggers for calciphylaxis. In addition to chronic kidney disease and hemodialysis, calciphylaxis could be associated with numerous non-uremic causes (malignancy, hyperparathyroidism, liver disease, skin trauma, autoimmune disorders, certain drugs, and many others). In liver dysfunction, calciphylaxis might be triggered by decreased levels of coagulation inhibitors, decrease of a circulating inhibitor of vascular ossification–calcification and vitamin K deficiency. Even chronic inflammatory states are possible risk factors since TNF-alpha was shown to induce osteogenic phenotype of human smooth muscle cells [9–11]. Although we have not found a complete explanation for the observed extensive and accelerated formation of small vessels' calcifications, we conclude that in the presented case, besides end-stage renal failure, hepatic impairment may certainly have played an important role [12].

Since we did not have precise information on stem cell type and origin, nor the circumstances in which the cell product was applied, we did not make further analyses and interpretations, in order to avoid any speculation. Nevertheless, the patient demanded discharge from the hospital due to fear at the growing scale of the COVID-19 epidemic at the time.

In the present paper, we therefore want to draw attention to two possible major risks of stem cell treatments operating with limited or no regulation: firstly, the possibility of severe multisystem side effects, including severe cardiomyopathy, liver and skin injury, and, secondly, the devastating socio-economic impact on the patient, leaving him financially strapped, with no physical improvement in his condition and no way of reclaiming losses.

## Conclusions

Unproven stem cell treatments may involve serious health, personal, and financial considerations. Regulatory agencies are expected to implement a comprehensive policy and measures clearly to delineate unproven stem cell therapies, which are putting increasing numbers of patients at risk. In cases in which there is a significant potential risk, probably the most effective way to combat this issue is to empower patients with the right knowledge on possible side effects, so allowing them to make an informed decision, and prevent further black scenarios from taking place.

## Supplementary Information

**Additional file 1: Table S1.** Results of important microbiology, metabolic and serology tests.

## Data Availability

Not applicable.

## Abbreviations

DM: Diabetes mellitus
GVHD: Graft versus host disease
