# Supplementary material for: Multiorgan failure with fatal outcome after stem cell tourism
Source: Eur J Med Res. 2021 Jan 9;26:5. doi: 10.1186/s40001-020-00477-4 (PMC7796809; doi:10.1186/s40001-020-00477-4)
Supplement: Supplementary file 1 — Additional file 1: Table S1. Results of important microbiology, metabolic and serology tests. [file 40001_2020_477_MOESM1_ESM.docx]

**Table S1** Results of important microbiology, metabolic and serology tests.

| MICROBIOLOGY/IMMUNOLOGY EXAMINATION | FINDING |
| --- | --- |
| CMV and EBV PCR (serum) | negative |
| Beta D glucan (serum) | negative |
| Aspergilus galactomanan antigen (ELISA,serum) | negative |
| Haemocultures (aerobic and anaerobic, serum) | negative in more instances |
| Biopsy of skin and subcutaneous tissue (leg) | both aerobic and anaerobic cultures negative |
| Central venous cathether exit site smear | negative |
| Central venous catheter tip | negative |
| Urine culture | negative |
| Nasopharyngeal swab SARS-CoV-2, influenza and respiratory syntitial virus | negative |
| Hepatitis B and C, HIV | negative |
| Skin wound swab (thorax)  Skin wound swab (right leg) | Staphylococcus aureus+++ Staphylococcus epidermidis+++ |
| IgG4 (serum) | 0,15 g/l (normal) |
| ANCA PR3 and MPO (serum, ELISA) | negative |
| Complement fractions C3 and C4 | normal |
| Antinuclear antibodies AND extractable nuclear antigen (ENA) panel, including anti-sM | negative |
| CERULOPLASMIN (SERUM) | normal |
